# Combined computational approaches for developing new anti-Alzheimer drug candidates: 3D-QSAR, molecular docking and molecular dynamics studies of liquiritigenin derivatives

**DOI:** 10.1016/j.heliyon.2022.e11991

**Published:** 2022-12-06

**Authors:** Hassan Nour, Ossama Daoui, Oussama Abchir, Souad ElKhattabi, Salah Belaidi, Samir Chtita

**Affiliations:** a Laboratory of Analytical and Molecular Chemistry, Faculty of Sciences Ben M'Sik, Hassan II University of Casablanca, Casablanca, 7955, Morocco; b Laboratory of Engineering, Systems and Applications, National School of Applied Sciences, Sidi Mohamed Ben Abdellah-Fez University, BP 72, Fez, Morocco; c Group of Computational and Medicinal Chemistry, LMCE Laboratory, University of Biskra, BP 145, Biskra 707000, Algeria

**Keywords:** Alzheimer's disease, Butyrylcholinesterase, Liquiritigenin, CoMFA, CoMISA, Molecular docking, Molecular dynamics

## Abstract

Butyrylcholinesterase is an acetylcholine-degrading enzyme involved in the memorization process, which is becoming an interesting target for the symptomatic treatment of Alzheimer's disease. In the present investigation, the structure–activity relationship of a set of Liquiritigenin derivatives recently revealed to be Butyrylcholinesterase inhibitors was studied basing on comparative field analysis (CoMFA) and comparative molecular similarity indices analysis (CoMISA). As a result, performant models with high predictive capability have been developed (CoMFA model: R^2^ = 0.91, Q^2^ = 0.62, R^2^_pred_ = 0.85; CoMISA model: R^2^ = 0.92, Q^2^ = 0.59, R^2^_pred_ = 0.83) and implemented to design new Liquiritigenin derivatives with improved activity. Besides, the affinity of the designed derivatives towards the active site of Butyrylcholinesterase, was confirmed by molecular docking and molecular dynamics studies. Moreover, they exhibited good pharmacokinetics properties. Accordingly, the outcomes of the present investigations can provide important direction for the development of new anti-Alzheimer's drug candidates.

## 1 Introduction

Loss of communication, learning or reasoning skills are among the common symptoms characterizing the development of Alzheimer's disease (AD) [1, 2]. Indeed, AD is a progressive and irreversible impairment of neurons, leading to the destruction of cognitive abilities [3]. In 2019, AD has been considered among the eight frequent causes of death worldwide [4]. Moreover, the World Health Organization predicted that in the coming years, there would be an increase in the number of patients suffering from neurodegenerative disorders [5]. The cholinergic hypothesis states that a decrease in the amount of acetylcholine, a neurotransmitter having a relevant role in memory processes, is the primary cause of the cognitive impairment seen as Alzheimer's disease progresses [6].

Butyrylcholinesterase (BuChE) is an enzyme that catalyzes the hydrolysis of acetylcholine into acetate and choline, further contributing to perturb the neurotransmission mechanisms [7, 8].

Therefore, reducing BuChE activity is an effective method for treating AD symptoms. Currently, the drugs used for the symptomatic treatment of AD act by limiting the hydrolysis of synaptic acetylcholine [9, 10]. This approach has proven to be effective in improving the intellectual functions of patients suffering from AD [11, 12]. However, the used drugs cause various adverse effects including hepatotoxicity, vomiting, fatigue, muscle cramps [13, 14, 15]. Therefore, the discovery of new drug candidates for the treatment of Alzheimer's disease is highly desired.

The current investigation attempts to explore the quantitative structure-activity relationship (QSAR) of a series of Liquiritigenin derivatives recently evaluated as Butyrylcholinesterase (BuChE) inhibitors [16], by applying Comparative Molecular Field Analysis (CoMFA) and Comparative Molecular Similarity Indices Analysis (CoMSIA) techniques. Besides, the developed models are applied to design new Liquiritigenin derivatives with improved activity. Moreover, the designed molecules are subjected to pharmacokinetics properties analysis, molecular docking, and molecular dynamics studies to study their drug-likeness properties, their affinity towards the BuChE enzyme and to study the stability of their complexes with the target receptor, respectively.

## 2 Materials and methods

### 2.1 Dataset collection and optimization

A set of thirty-three liquiritigenin derivatives acting as Butyrylcholinesterase inhibitors was retrieved from the literature [16]. The collected database was first randomly partitioned into a training set (which includes 27 derivatives) and a test set (which includes 6 derivatives). Moreover, the half-maximal inhibitory concentration (IC_50_) values expressed in μM, quantifying the capacity of the studied derivatives in inhibiting the Butyrylcholinesterase enzymatic activity, were transformed into negative logarithm (pIC_50_ = −log (IC_50_) to be considered as dependent variable during the modeling process. The investigated structures as well as their corresponding pIC_50_ values are depicted in Table 1.

**Table 1:** Structures and anti-BuChE activities of studied molecules.

N°	Structure	pIC_50_	N°	Structure	pIC_50_	N°	Structure	pIC_50_
**3a**∗		5.020	**3k**		5.412	**4e**		5.123
**3b**∗		5.262	**3l**		5.388	**4f**		5.166
**3c**		5.396	**3m**		5.343	**4g**		5.151
**3d**		5.588	**3n**∗		5.466	**4h**		5.158
**3e**		5.270	**3o**∗		5.248	**4i**∗		5.412
**3f**		5.445	**3p**		5.299	**4j**		5.282
**3g**		5.375	**4a**		5.184	**4k**		5.219
**3h**		5.459	**4b**		5.132	**4l**		5.184
**3i**∗		5.299	**4c**		5.293	**4m**		5.239
**3j**		5.347	**4d**		5.165	**4n**		5.407
**4o**		5.263	**4p**		5.269	**Li**^a^		4.853

∗ Refer to test set molecules.

a Liquiritigenin.

Besides, all collected structures were optimized using the Powell conjugate gradient algorithm and Gasteiger-Huckel partial atomic charge, under the Tripos force field implemented in the SYBYL-X.2.1 software. The convergence criterion was set at 0.05 kcal/mol with 1,000 iterations [17, 18, 19, 20, 21, 22].

### 2.2 Molecular alignment

Molecular alignment is an extremely important procedure in 3D-QSAR analysis. Therefore, before starting modeling, the most active compound (3d) (pIC_50_ = 5.59) was chosen as template structure, and then all remaining molecules were subjected to a molecular alignment process, based on their structural similarities to the template skeleton, by using the distill module available in SYBYL 2.1 software [17].

### 2.3 QSAR modeling

To identify the most important structural properties governing the biological activity of the studied compounds, 3D-QSAR models were developed by adopting Comparative Molecular Field Analysis (CoMFA) and Comparative Molecular Similarity Indices Analysis (CoMSIA) techniques [23, 24]. These techniques allow us to explore the effects of different molecular descriptors, including steric, electrostatic, hydrophobic, donor of hydrogen bonds and acceptor of hydrogen bonds, on biological activity [25]. In the current study, the quantitative relationship between 3D field descriptors and anti-Butyrylcholinesterase activity (pIC_50_) was correlated by applying the partial least squares (PLS) algorithm in the Tripos force field (reference input lattice defined at 2 Å in all cartesian directions, sp^3^ hybrid carbon atom as the source for steric and electrostatic energy calculations, default cutoff energy setting at 30 kcal/mol) [26, 27]. Further, suitable QSAR models describing the structure-activity relationship of studied derivative were selected based on various statistical significance criteria including coefficient of determination (R^2^), standard error of estimate (SEE), coefficient of determination by cross-validation (Q^2^_loocv_), Fischer's test (F), coefficient of determination calculated for the test set (R^2^_pred_), and Y-randomization test [28, 29, 30].

### 2.4 Model application and docking studies

By exploiting the developed models, new molecules with enhanced activities have been designed. Further, molecular docking studies were carried out using the Autodock Vina program [31] to investigate the mode of interaction involved between the designed molecules and the target protein as well as to evaluate their affinity towards the active site of the Butyrylcholinesterase receptor (BuChE). The crystal structure of Butyrylcholinesterase (PDB ID: 6EUL) was download from the RCSB Protein Data Bank available online at (www.RCSB.org/structure/6EUL). The following procedure was adopted to prepare the target protein structure:

1. Crystallized ligands and water molecules were taken out of the protein structure.
2. Polar hydrogens and Kollman charges were added.
3. A docking gird box with a size of 40 × 40 × 40 Å^3^ and spacing of 0.375 Å between its points, was created to perform the docking study. The coordinates x = 42.834, y = 19.853, z = 24.398 were defined as the gird center covering the binding site of BuChE.
4. The PDBQT file corresponding to the prepared protein was generated.

Furthermore, the MMFF94 force field and the steepest Descent method (5,000 steps) implemented in the Avogadro program were used to optimize the ligand structures [32, 33, 34, 35, 36, 37]. After finishing the optimization, the polar hydrogens were merged, Gasteiger charges were added, and the PDBQT files relative to the optimized ligands were generated using AutoDockTools [38]. The prepared ligands were docked to the target protein, and then, the implicated interactions were analyzed using the software Discovery Studio 2021 [39].

### 2.5 Molecular dynamics simulation

Molecular dynamics simulations (MD) were carried out using GROMACS software with the charmm27force field [40, 41, 42, 43, 44], to study the stability of the interactions involved between BuChE and the best docked ligand in terms of binding affinity. The topology corresponding to the studied ligand was prepared using Swiss Param server [45]. Besides, the studied system was delimited by a cubic simulation box and then solvated by using TIP3P water molecules. To neutralize the studied system, two chloride ions were added. Following that, the simulated system was optimized using steepest descent minimization to avoid steric clashes. Then, the system undergone NVT equilibration by adopting V-rescale thermostat at 300k during 1ns [46], and then NPT equilibration was performed utilizing Parrinello-Rahman barostat at 1 atm for 1 ns [47], to stabilize the system at the desired conditions. Finally, the equilibrated system was subjected to MD 100 ns. From the simulation results, we calculated various parameters e.g., solvent Accessible Surface Area (SASA), root mean square deviation (RMSD), Radius of gyration (Rg), number of hydrogen bonds, and root mean square fluctuation (RMSF).

## 3 Results and discussion

### 3.1 Molecular alignment Protocol

**Figure 1:**
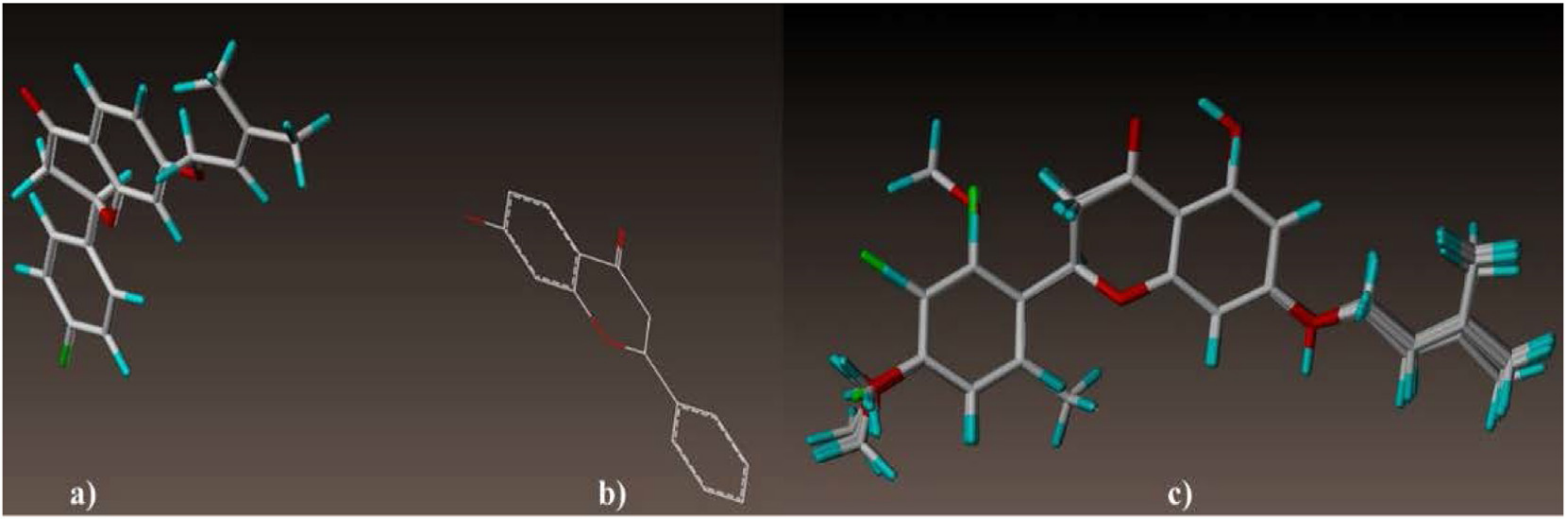
(a) Template structure (3d), (b) common core, (c) molecular alignment set.

Molecular alignment is an essential pre-procedure to any CoMFA and CoMSIA analysis. In the current study, the 3D molecular structures corresponding to the liquiritigenin derivatives were optimized and then aligned based on their structural similarities to the template skeleton (3d) shown in Figure 1a. As illustrated in Figure 1c, all the investigated molecular structures are properly aligned with the template structure. The common core of all superposed molecules is presented in Figure 1b.

### 3.2 Partial least square analysis

PLS analysis was performed to explore the quantitative relationship between pIC_50_ and the 3D field descriptors computed via CoMFA and CoMSIA techniques. Table 2 provides the statistical indices values for the best developed models conforming to Golbraikh and Tropsha's criteria [28, 29].

**Table 2:** Statistical metrics of proposed CoMFA and CoMSIA models.

	Q^2^	R^2^	SEE	F	N	R^2^_pred_	Fractions				
							S	E	H	D	A
CoMFA	0.62	0.91	0.057	12.76	5	0.85	0.291	0.709	–	–	–
CoMSIA	0.59	0.92	0.051	12.62	5	0.83	0.064	0.278	0.154	0.072	0.432

Q^2^ = coefficient of cross-validation correlation; N = Optimal number of components identified by Leave-One-Out Cross-Validation (loocv); SEE = Standard Error of Estimate; R^2^ = Conventional coefficient of determination; R^2^_pred_ = Coefficient of determination according to external test; SEE = Standard Error of Estimate; Fractions: Contributions of steric (S), electrostatic (E), hydrophobic (H), donor (D) and acceptor (A) hydrogen bonds.

**Figure 2:**
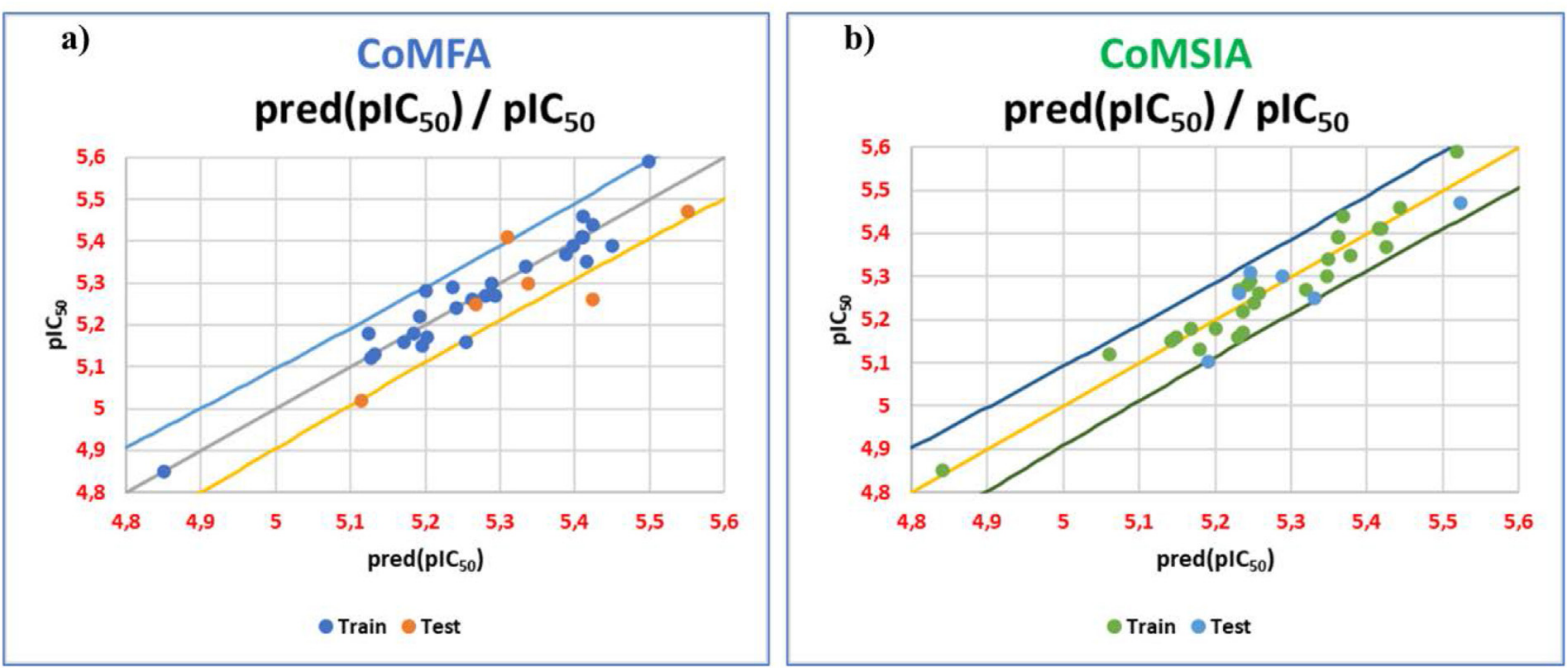
Correlation between the observed activities and those predicted by CoMFA (a) and CoMSIA (b) models.

From Table 2, we can notice that the statistical significance indices for CoMFA and CoMSIA are strongly convergent, indicating that there is a strong correlation between the biological inhibitory activity (pIC50) of BuChE and five descriptors, namely steric, electrostatic, hydrophobic, and hydrogen bonds fields. Additionally, the high R^2^_pred_ parameter values (0.85 and 0.83) achieved from external validation tests reveal the strong predictive ability of our developed models. The high predictive power of the two models proposed via CoMFA and CoMSIA techniques can be further confirmed by the very low residual values observed between the *in vitro* observed pIC_50_ and the *in silico* predicted pIC_50_ (Table 3), as well as by the linear and uniform distribution of observed pIC_50_ values versus *in silico* calculated values (Figure 2-a and Figure 2-b).

**Table 3:** Values of pIC_50_ observed and predicted by CoMFA (a) and CoMSIA (b) models.

			CoMFA		CoMSIA	
	Compound	BuChE pIC_50_	predicted (pIC_50_)	Residuals	predicted (pIC_50_)	Residuals
**Training set**	3c	5.39	5.450	−0.06	5.356	0.034
	3d	5.59	5.498	0.092	5.507	0.083
	3e	5.27	5.294	−0.024	5.314	−0.044
	3f	5.44	5.424	0.016	5.362	0.078
	3g	5.37	5.388	−0.018	5.417	−0.047
	3h	5.46	5.41	0.05	5.434	0.026
	3j	5.35	5.415	−0.065	5.372	−0.022
	3k	5.41	5.409	0.001	5.407	0.003
	3l	5.39	5.398	−0.008	5.356	0.034
	3m	5.34	5.334	0.006	5.343	−0.003
	3p	5.3	5.289	0.011	5.341	−0.041
	4a	5.18	5.125	0.055	5.168	0.012
	4b	5.13	5.132	−0.002	5.178	−0.048
	4c	5.29	5.236	0.054	5.243	0.047
	4d	5.16	5.254	−0.094	5.227	−0.067
	4e	5.12	5.128	−0.008	5.063	0.057
	4f	5.17	5.203	−0.033	5.234	−0.064
	4g	5.15	5.196	−0.046	5.142	0.008
	4h	5.16	5.172	−0.012	5.148	0.012
	4j	5.28	5.201	0.079	5.241	0.039
	4k	5.22	5.193	0.027	5.234	−0.014
	4l	5.18	5.184	−0.004	5.2	−0.02
	4m	5.24	5.241	−0.001	5.248	−0.008
	4n	5.41	5.41	0	5.41	0
	4°	5.26	5.263	−0.003	5.255	0.005
	4p	5.27	5.28	−0.01	5.229	0.041
	liquiritigenin	4.85	4.851	−0.001	4.85	0
**Test set**	3a	5.02	5.114	−0.094	5.189	−0.169
	3b	5.26	5.4234	−0.1634	5.229	0.031
	3i	5.3	5.338	−0.038	5.285	0.015
	3n	5.47	5.551	−0.081	5.511	−0.041
	3°	5.25	5.267	−0.017	5.325	−0.075
	4i	5.41	5.309	−0.06	5.243	0.167

Besides, although the calculated internal and external validation parameters indicate the validity of the CoMFA and CoMSIA models, it is necessary to test the hypothesis of chance involved in the strong structure-activity relationship. For this reason, Y-randomization test was performed to assess the stability level of linear relationship between the structures of liquiritigenin derivatives and their anti-BuChE activity (Table 4).

**Table 4:** Y-randomization test parameters.

	CoMFA			CoMSIA/SEHDA		
Iteration	Q^2^_yrand_	R^2^_yrand_	^c^R^2^_p_	Q^2^_yrand_	R^2^_yrand_	^c^R^2^_p_
Original	0.62	0.91	–	0.59	0.92	–
01	0.328	0.417	0.667	0.497	0.513	0.606
02	0.216	0.474	0.627	0.328	0.278	0.761
03	−0.11	0.538	0.579	−0.624	0.511	0.608
04	−0.245	0.387	0.687	0.403	0.403	0.683
05	−0.447	0.535	0.582	−0.536	0.326	0.732
Average	−0.052	0.470	0.629	0.014	0.406	0.678

From the Y-randomization test scores presented in Table 4, we can notice that R^2^ and Q^2^ values of CoMFA and CoMSIA random models are lower than those of the original models. In addition, the average ^c^R^2^_p_ values are inferior to 0.5, which means that the high correlation level between pIC_50_ and the structure of liquiritigenin derivatives obtained via the CoMFA and CoMSIA models is not due to coincidence. Thus, the developed models can be applied with confidence to design new Liquiritigenin derivatives with improved anti-BuChE activity.

### 3.3 Three-dimensional analysis of CoMFA and CoMSIA contour maps

**Figure 3:**
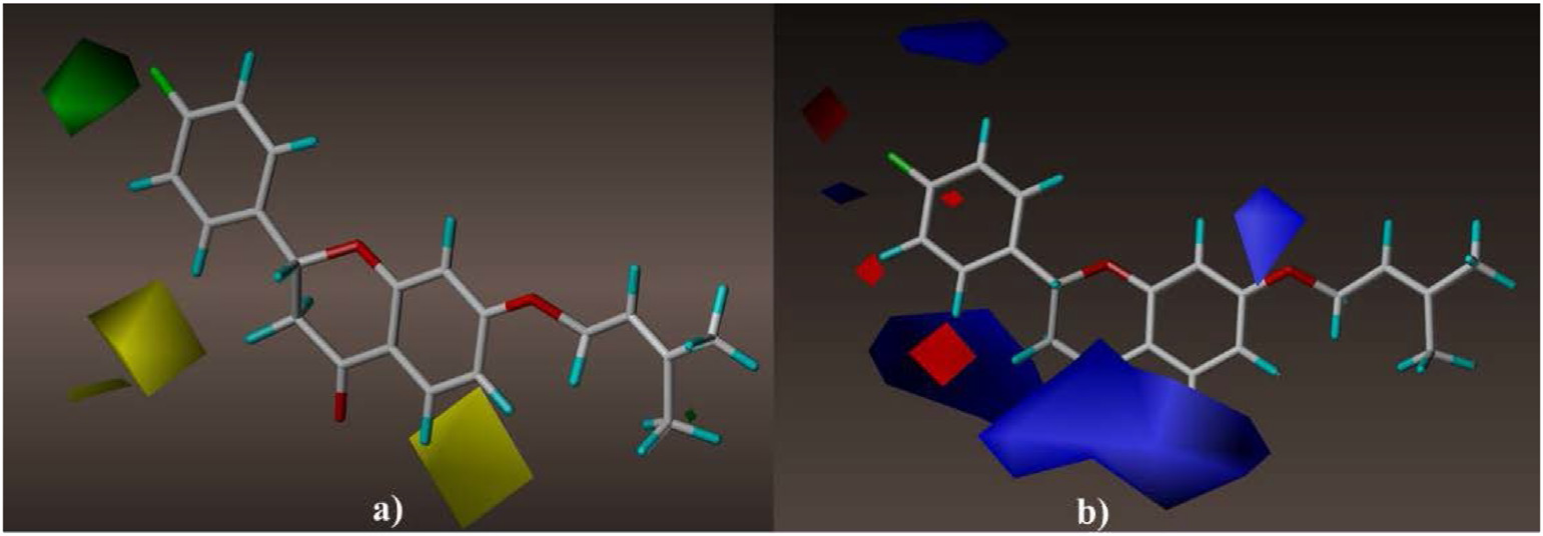
Contour maps generated by CoMFA model, (a) steric field interactions (green = favorable/yellow = unfavorable), (b) electrostatic field interactions (blue = favorable/red = unfavorable).

**Figure 4:**
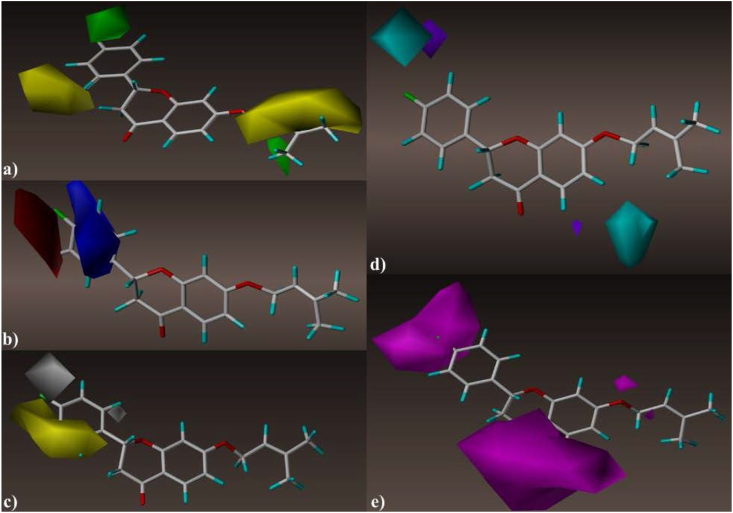
Contour maps generated by CoMSIA model, (a) steric field interactions (green = favorable/yellow = unfavorable), (b) electrostatic field interactions (blue = favorable/red = unfavorable), (c) hydrophobic field interactions (yellow = favorable/white = unfavorable), (d) hydrogen bond-donor field interactions (cyan = favorable/purple = unfavorable), (e) hydrogen bond-acceptor field interactions (magenta = favorable/red = unfavorable).

The contour maps of QSAR models are visual representations of the structure-activity relationship corresponding to a given series of chemicals. In the current study, the contour maps relative to the developed models were analyzed, to identify the requirements influencing the anti-BuChE activity. The three-dimensional analysis of these graphical visualizations leads to identify regions in the template molecule requiring appropriate modifications to design new molecules with improved biological activity compared to the investigated derivatives. Figures 3 and 4 highlight the visualizations of contour maps in 3D space based on the structure of the template molecule (3d) generated using CoMFA and CoMSIA models, respectively.

**Figure 5:**
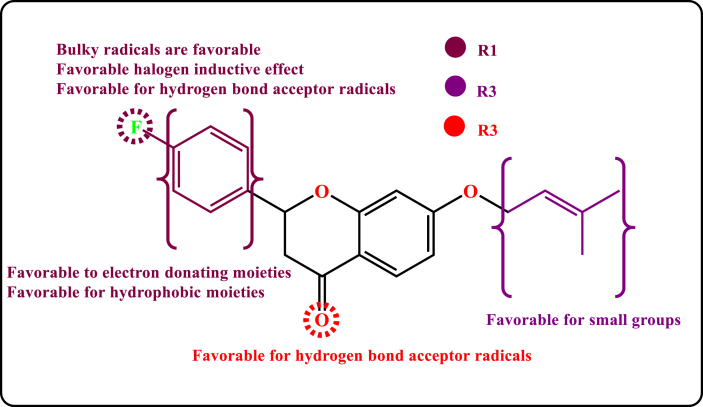
Structural properties characterization favorable to design powerful BuChE inhibitors.

The reinforcement of the fluorobenzene (R1) group by bulky radicals at the para site is beneficial for boosting the biological inhibitory activity against BuChE (Figures 3a and 4a), as can be seen from the visualizations of the contour maps produced by the established models (CoMFA [Figure 3] and CoMSIA [Figure 4]). This can be confirmed from Table 1, when a hydrogen atom in molecule 3a (pIC_50_ = 5.02) was substituted by a fluorine atom in para site, the biological activity of molecule 3d improved (pIC_50_ = 5.59). The yellow contours near the ortho, para sites in the phenyl ring and also near **R2** ((3-methylbut-2-en-1-yl) oxy) moiety indicate that these areas are not suitable for large radicals (Figure 4a). Regarding the effect of electrostatic field, we can observe that blue contours are more abundant than red ones around the structure of the template molecule (3d) (Figure 3b). This means that the insertion of electron donating moieties into the structure of molecule 3d may be favorable for improving the biological activity. While we note that the benzene ring is surrounded by the blue contour in Figure 4b, this means that the enhancement of this region by electron donating elements leads to improved anti-BuChE activity. On the other hand, the presence of the red outline near the meta, para sites in the phenyl ring means that reinforcement of these sites with additional electron-accepting elements such as halogens is favorable for enhancing pIC_50_ biological inhibitory activity (Figure 4b). Regarding the effects of hydrophobic field (Figure 4c), we can observe the abundance of yellow contours compared to white at the fluorobenzene group, it means that the enhancement of this region by hydrophobic groups is favorable for the improvement of the target biological activity. Besides, we note that the hydrogen bond donor contour maps shown in Figure 4e are more generously localized on the structure of the template molecule (3d) compared to the H-bond donor contour mapping presented in Figure 4d. This can be confirmed by Table 2, the contribution of descriptor D (0.072) is very low compared to the contribution of descriptor A (0.432). Thus, the insertion of hydrogen bond acceptor moieties in the para position belonging to phenyl ring (**R1**) and in the chroman moiety (**R3**) is favorable for improving the biological activity of liquiritigenin derivatives against Alzheimer's disease. Figure 5 presents the rationalized positions on the structure of the template molecule (3d) and their favorable spatial properties leading to improved biological activity of liquiritigenin derivatives against Alzheimer's through inhibition of Butyrylcholinesterase.

### 3.4 Design of new BuChE inhibitors

The descriptors influencing the anti-Butyrylcholinesterase activity mentioned above have been considered to design novel compounds with increased activity. Table 5 presents their structures as well as their activity values (pIC_50_) predicted by the developed models.

**Table 5:** Structures of new designed compounds as well as their pIC50 values predicted by CoMFA and CoMSIA models.

		3D-QSAR models	
Name	Structures	CoMFA pred(pIC_50_)	CoMSIA pred(pIC_50_)
L1		5.556	5.993
L2		5.553	5.964
L3		5.545	5.887
L4		5.542	5.839
L5		5.652	5.824
L6		5.544	5.865
L7		5.612	6.107
L8		5.725	6.327
L9		5.457	5.792
L11		5.454	5.732
L12		5.593	5.784
L13		5.642	6.001
L14		5.753	6.425
L15		5.754	6.507
L16		5.612	6.102
L17		5.633	6.211
L18		5.629	6.137
L19		5.632	5.641

### 3.5 Molecular docking study

The obtained results, which are presented in Table 6, show that the docked ligands' binding energies are negatives. Thus, complexes with favorable thermodynamic properties are produced through molecular docking between the designed chemicals and their target protein. Additionally, all docked ligands display low binding energies comparable to that of Rivastagmine, an FDA-approved medication for Alzheimer's disease, demonstrating their significant affinity towards the BuChE protein and corroborating their strong anti-BuChE activities predicted by the developed QSAR models.

**Table 6:** Binding energies for docked ligands.

Ligand	Binding energy (kcal/mol)
**L 7**	−8.8
**L 8**	−9.0
**L 13**	−8.5
**L 14**	−9.2
**L 15**	−8.8
**L 16**	−9.9
**L 17**	−9.0
**L 18**	−9.2
Rivastagmine	−7.3

**Figure 6:**
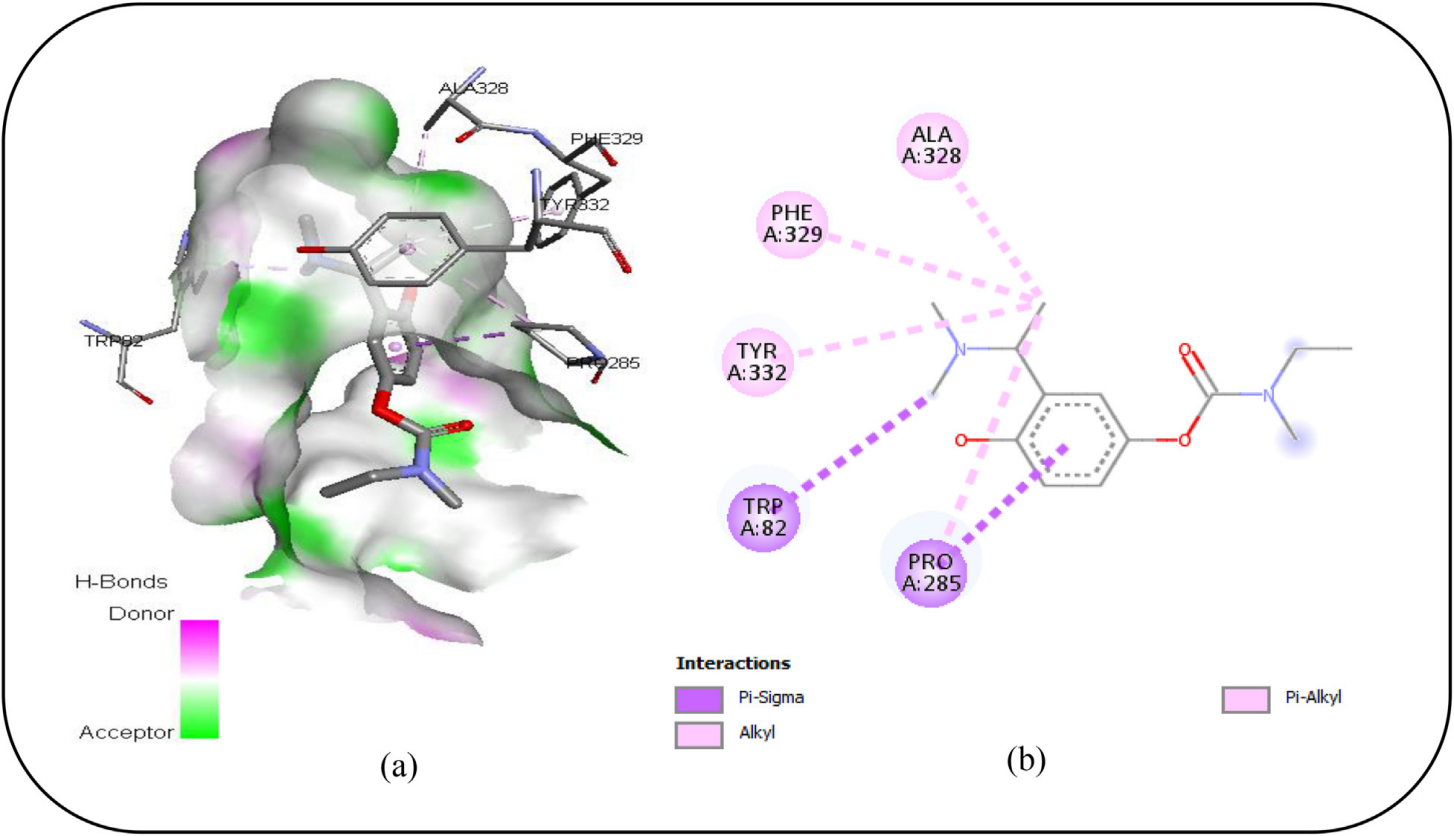
(a) 3D and (b) 2D diagram revealing the interactions between Rivastigmine and BuChE receptor.

Besides, to identify the active sites impacting the anti-BuChE activity, the interactions implicated between BuChE and rivastigmine (Figure 6a and b) were analyzed. As depicted in Figure 6a and b, Rivastigmine binds to its respective receptor through various interactions, including Pi-sigma and Alkyl interactions, with TRP A: 82, PRO A: 285, ALA A: 328, PHE A: 329 and TYR A: 332. Therefore, these residues can be identified as the most powerful active sites that drive the BuChE enzymatic activity.

**Figure 7:**
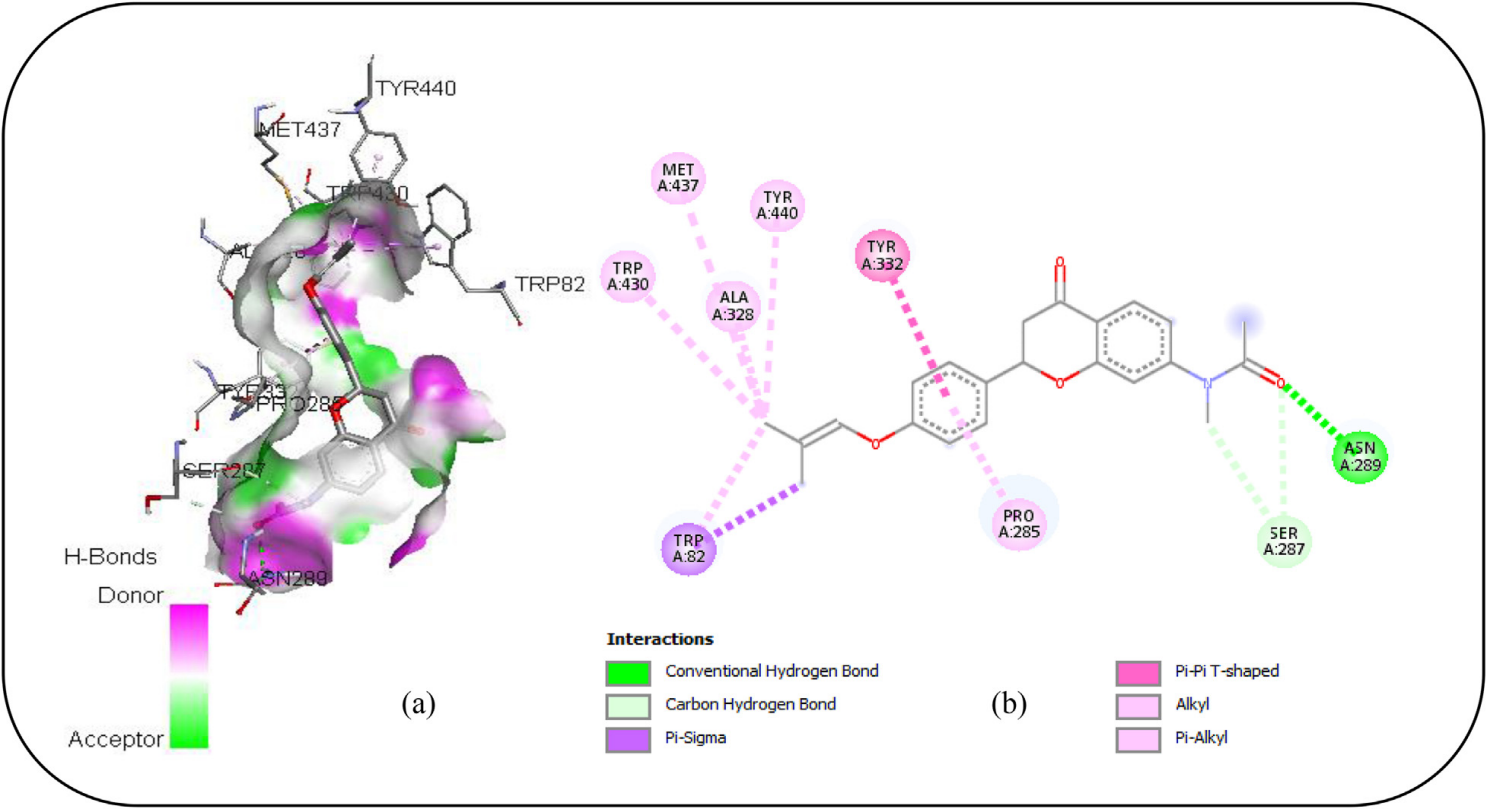
(a) 3D and (b) 2D diagram revealing the interactions involved between L16 and BuChE receptor.

From Table 6, L16 exhibited the highest binding affinity towards the target protein (BuChE). To gain insights into its mode of interaction with BuChE, the involved interactions were analyzed. As depicted in Figure 7a and b, the ligand (L16) having exhibited the highest binding affinity towards BuChE involves various molecular interactions to form a coherent complex with the target biological receptor. Indeed, it turned out that it implicates six Alkyl interactions with TYR A: 440, MET A: 437, ALA A: 328, TRP A: 430, TRP A:82 and PRO A:285, three Hydrogen bonds with ASN A: 289 and SER A:287, as well as a Pi-Sigma interaction with TRP A:82 and a Pi-Pi T-shaped interaction with TYR A: 332. Furthermore, L16's backbone is well covered by these interactions. Additionally, prior investigations have shown that the residues with which L16 interacted are some of the important sites controlling the inhibition of BuChE [48, 49].

In conclusion, the inhibitory impact of L16 against the Butyrylcholinesterase protein was strengthened by interactions with specific active sites that are responsible for the targeted protein's enzymatic activity.

### 3.6 MD simulations

**Figure 8:**
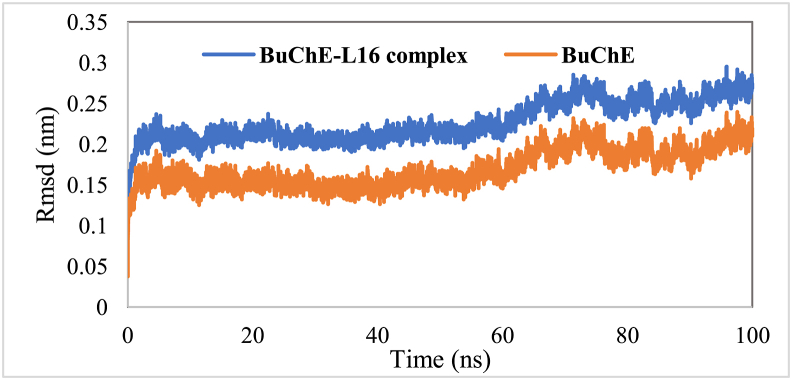
Root mean square deviation (RMSD) of backbone of BuChE and BuChE- L16 as function of time.

To gain insight into the influence of L16 on the structural stability of BuChE, the dynamic behavior of BuChE uncomplex and its complex with L16 was simulated in aqueous environment during 100 ns. As shown in Figure 8, the RMSD of all systems was in steady state between 0 and 60 ns, and then exhibited negligible fluctuation between 60 ns and 70 ns followed by a second steady state. Moreover, the studied systems (complex and BuChE uncomplex) displayed similar RMSD profiles, demonstrating that L16 did not induce any noticeable conformational changes in the structure of BuChE during the simulation. Besides, we note that the RMSD values corresponding to the investigated systems do not exceed 0.25 nm during the entire simulation. In addition, 0.169 and 0.226 nm were predicted to be the RMSD average values of BuChE and its complex, respectively. RMSD below 0.3 nm is appreciable and within 0.5 nm is considered to be in an acceptable range [50]. Therefore, the RMSD values exhibited by BuChE and its complex with L16 indicate no significant changes, conferring durable stability of BuChE-L16 complex in an aqueous medium.

**Figure 9:**
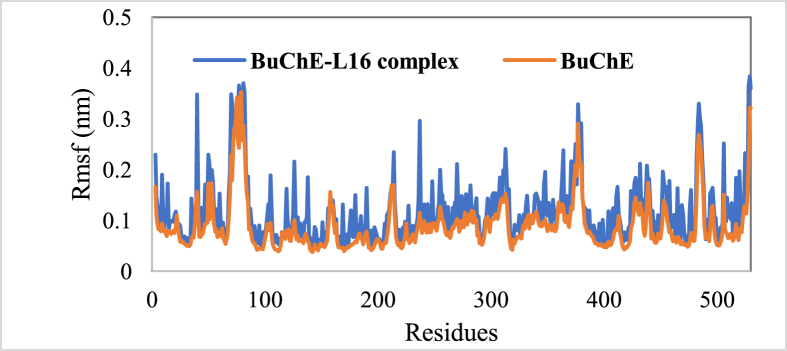
Root mean square fluctuation (RMSF) of Cα atoms of BuChE in the absence and presence of L 16.

Further, the stability of the systems under investigation was also assessed by examining the variance of their root means square fluctuation (RMSF) as illustrated in Figure 9. The RMSF average of BuChE and BuChE-L16 were found to be 0.089 nm and 0.117 nm, respectively. In addition, the majority of amino acid residues belonging to BuChE and BChE-L16 complex are below 0.3 nm. Overall, the RMSF graph illustrates that there are no significant alterations in the residual fluctuations upon binding of L16 with BuChE.

**Figure 10:**
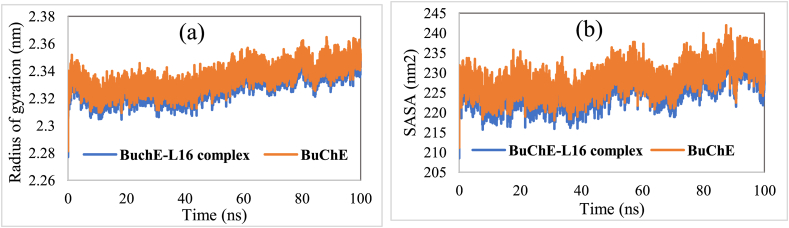
(a) Radius of gyration (Rg) of BuChE and BuChE-L16 complex as function of time. (b) Solvent accessible surface area (SASA) of BuChE and BuChE-L16 complex.

Radius of gyration (Rg) is an important parameter to evaluate the stability of proteins in MD simulations [48]. Figure 10(a) illustrates the Rg graphs related to the studied systems. The Rg of all systems were relatively at steady state during the simulation period, highlighting the structural dynamics stability of the studied systems. 2.334 and 2.329 nm were predicted to be the average Rg values for BuChE and BuChE-L16, respectively. Besides, the solvent accessible surface area (SASA) describing the protein surface area variation during a simulation period was also calculated and presented in Figure 10(b). According to the obtained results, 228.266 and 226.209 nm^2^ were predicted to be the average SASA values of BuChE and BuChE-L16, respectively. Furthermore, it's clear that the Rg trajectory remained relatively stable during the entire simulation, illustrating the investigated systems' subtilty in aqueous medium. Overall, the analyzed Rg and SASA graphs suggesting that the systems did not undergo any noticeable conformational changes during the entire simulation.

**Figure 11:**
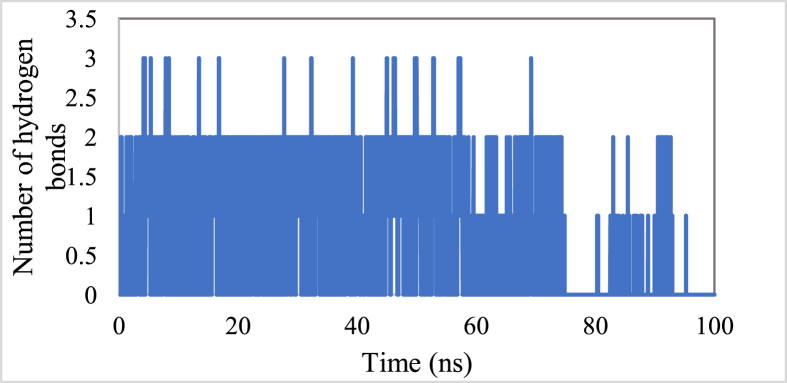
Number of hydrogens implicated between L16 and BuChE as function of time.

The hydrogen bonds implicated between L16 and BuChE throughout the simulation period, were also predicted (Figure 11). As evident from Figure 11, L16 was found to be consistently hydrogen bound to BuChE during the whole simulation, supporting the stability of the BuChE- L16 complex.

### 3.7 *In silico* pharmacokinetics analysis

Drug candidates can fail during preclinical or clinical trials due to poor pharmaceutical properties, which can delay drug development and waste implicated resources. Accordingly, *in silico* evaluating the pharmacokinetic properties of new drug candidates it is of great importance. In the current study, the drug-likeness properties and the potential toxicity risks corresponding to the designed compounds having exhibited the highest predicted activity were evaluated using the pKCSM online server. In addition, their ADME properties were predicted using SwissADME server.

As presented in Table 7, all evaluated molecules are likely to be bioavailable in the human body, favoring their use as oral drug. Indeed, according to Lipinski and Veber rules, a drug candidate having a molecular weight (MW) less than 500 Daltons, number of hydrogen bond acceptor not exceeding 10, no more than five hydrogen bond donors, coefficient of Octane-water partition (log P) not exceeding 5, no more than ten rotatable bonds and polar surface area less than 140 Å^2^, is likely to be bioavailable as long as it does not violate more than one of the cited parameters. Besides, the toxicity analysis revealed that no evaluated molecule is likely to be mutagenic or irritant and does not lead to acquire long QT (Table 8). Furthermore, no compound is hepatotoxic, and they may dangerous only at very high doses. In addition, as presented in Table 9, all investigated compounds are predicted to be highly absorbed through the human intestine and likely to cross the blood-brain barrier (BBB), supporting their use as anti-Alzheimer drug.

**Table 7:** Drug-likeness parameters according to Lipinski and Veber rules.

Compound	Lipinski and Veber rules						Number of violations
	MW	Log P	RB	HBA	HBD	PSA	
**L7**	286.302	3.931	3	3	0	121.862	0
**L8**	272.275	3.541	2	3	0	115.497	0
**L13**	298.338	3.800	4	4	0	129.174	0
**L14**	283.327	3.443	3	4	1	123.246	0
**L15**	311.381	3.858	4	4	0	136.186	0
**L16**	365.429	4.678	4	4	0	158.752	1
**L17**	337.375	3.898	4	4	0	146.023	1
**L18**	339.391	3.774	4	4	0	146.712	1
Threshold							
MW ≤ 500		LogP ≤ 5	RB ≤10	HBA ≤ 10	HBD ≤ 5	PSA ≤140	N. Viol ≤ 1

**Table 8:** Toxicity risks of designed compounds predicted by pKCSM server.

Comp	Toxicity parameters							
	AMES toxicity	Max. tolerated dose (human)	hERG I inhibitor	hERG II inhibitor	LD50	LOAEL	Hepatotoxicity	Skin Sensitisation
L 7	No	0.744	No	No	2.513	2.016	No	No
L8	No	0.708	No	No	2.466	1.994	No	No
L13	No	0.704	No	No	2.533	2.052	No	No
L14	No	0.409	No	No	2.019	2.100	No	No
L15	No	0.569	Yes	No	2.503	1.768	No	No
L16	No	0.782	No	No	2.233	1.829	No	No
L17	No	0.774	No	No	2.141	1.859	No	No
L18	No	0.412	No	Yes	2.626	1.637	No	No

**Table 9:** ADME properties of designed compounds.

Comp	ADME properties							
	GI absorption	BBB permeant	P-gp substrate	CYP1A2 inhibitor	CYP2C19 inhibitor	CYP2C9 inhibitor	CYP2D6 inhibitor	CYP3A4 inhibitor
L7	High	Yes	No	Yes	Yes	No	Yes	Yes
L8	High	Yes	No	Yes	Yes	No	Yes	Yes
L13	High	Yes	No	Yes	Yes	Yes	Yes	Yes
L14	High	Yes	Yes	Yes	Yes	No	Yes	Yes
L15	High	Yes	No	Yes	Yes	Yes	Yes	Yes
L16	High	Yes	No	No	Yes	Yes	Yes	Yes
L17	High	Yes	No	No	Yes	Yes	No	Yes
L18	High	Yes	No	No	Yes	Yes	Yes	Yes

P-gp is an ATP-binding cassette (ABC) transporter functioning as biological barrier by extruding toxins and xenobiotics out of cells. As presented in Table 9, except for L 14, no compound is likely to be P-gp substrate.

Cytochrome P450 is an important enzyme for the body's detoxification process and relevant for the metabolism of numerous medications. Based on the CYP1A2 isoform, L16, L17 and L18 are not likely to be cytochrome P450 inhibitors.

## 4 Conclusion

The present study revealed the quantitative structure-activity relationship of a series of Liquiritigenin derivatives using two techniques, namely Comparative Molecular Field Analysis (CoMFA) and Comparative Molecular Similarity Indices Analysis (CoMSIA). The developed models exhibited relevant statistical performances, notably in terms of predictivity and robustness (CoMFA model: R^2^ = 0.91, Q^2^ = 0.62, R^2^_pred_ = 0.85; CoMISA model: R^2^ = 0.92, Q^2^ = 0.59, R^2^_pred_ = 0.83). The contour maps related to the developed models indicate that the insertion of electron donating moieties, electron-accepting elements, hydrophobic groups, bulky radicals, or hydrogen bond acceptor moieties in specific regions belonging to the template structure (3d) leads to new Liquiritigenin derivatives with enhanced anti-BuChE activity. Taking into consideration the features governed the anti-BuChE activity, which have been described by the developed models, new Liquiritigenin derivatives with enhanced activities were designed. The designed compounds exhibited significant affinity towards the BuChE protein corroborating their strong anti-BuChE activities predicted by the developed QSAR models. The pharmacokinetics properties analysis, molecular docking and molecular dynamics studies confirmed that L16, new designed compound, can be promising drug candidate for the symptomatic treatment of AD. Indeed, L16 was predicted to be bioavailable in the human body, non-toxic, non-mutagenic and likely to form stable complex with BuChE in aqueous medium. Therefore, our developed models validated based on Golbraikh and Tropsha's criteria are highly recommended as guide for rational design of new anti-BuChE. Besides, we recommend the synthesis of L16 for future exploitation as promising BuChE inhibitor.

## Declarations

### Author contribution statement

Hassan Nour: Conceived and designed the experiments; Performed the experiments; Analyzed and interpreted the data; Wrote the paper.

Ossama Daoui, Oussama Abchir: Conceived and designed the experiments; Analyzed and interpreted the data; Wrote the paper.

Souad ElKhattabi, Salah Belaidi: Analyzed and interpreted the data; Contributed reagents, materials, analysis tools or data.

Samir Chtita: Conceived and designed the experiments; Performed the experiments; Analyzed and interpreted the data; Contributed reagents, materials, analysis tools or data; Wrote the paper.

### Funding statement

This research did not receive any specific grant from funding agencies in the public, commercial, or not-for-profit sectors.

### Data availability statement

Data included in article/supplementary material/referenced in article.

### Declaration of interests statement

The authors declare no conflict of interest.

### Additional information

No additional information is available for this paper.
